# World’s Fair Items

**Published:** 1892-05

**Authors:** 


### World's Fair Items.

Of the 75,560,000 feet of lumber required for the Exposition buildings, docks and electric subways, 54,875,800 have been placed. Of iron and steel 39,490,900 pounds, or nearly 20,000 tons, are required. Of this nearly half is in place, and the remainder will all be in position before the first of June.

